# The subtle science: a systematic review of psilocybin magic mushroom and truffle microdosing

**DOI:** 10.3389/fpsyt.2026.1866881

**Published:** 2026-08-19

**Authors:** Jeni Page, Alexandra Moore, Bridget E. Hawkins, Elizabeth J. Lyons, Elizabeth Lorenzo

**Affiliations:** 1 School of Nursing, Texas Tech University Health Sciences Center, Lubbock, TX, United States; 2 Cook Children’s Medical Center, Fort Worth, TX, United States; 3 School of Nursing, Research Innovation and Scientific Excellence Center, University of Texas Medical Branch, Galveston, TX, United States; 4 The University of Texas Medical Branch at Galveston School of Health Professions, Galveston, TX, United States; 5 The University of Texas Medical Branch at Galveston School of Nursing, Galveston, TX, United States

**Keywords:** magic mushrooms, microdosing, psilocybin, psychedelic substances, truffles

## Abstract

**Introduction:**

Microdosing psilocybin magic mushrooms (MM) and truffles has grown in popularity, but the evidence supporting reported benefits remains poorly characterized. The purpose of this systematic review was to synthesize the existing literature on microdosing psilocybin MM and truffles in adults to determine reported health effects and perceived benefits, motivations for use, dosing protocols and preparations, adverse events, and competing interests.

**Methods:**

This systematic review followed the Preferred Reporting Items for Systematic Reviews and Meta-Analyses (PRISMA) and was registered with the International Prospective Register of Systematic Reviews (PROSPERO; CRD42023428107). Studies of adults microdosing psilocybin MM or truffles were eligible. Risk of bias was assessed across included studies.

**Results:**

Eleven articles (five quantitative and six qualitative studies) met inclusion criteria. Studies were conducted in the United States (n=4), Netherlands, Argentina, and internationally (n=2 each), and Slovenia (n=1) with a total sample of 3,262 adults. Some studies reported microdosing psilocybin MM or truffles may improve well-being, mental health, cognition, physical health, creativity, relationships, pain, curiosity, and substance dependence, and enhance senses among adults. However, the majority of evidence had at least some concerns for risk of bias or was qualitative and used a variety of self-reported outcome measures. Approximately 86% of microdoser motivations were non-medical and only 14% were related to medical diagnoses, mostly for mental health improvements. Extreme heterogeneity was identified across dosing protocols and preparations. The majority of studies did not report adverse events but for those reported, they were mostly mild. All but four studies declared no conflicts of interest and four did not disclose funding sources.

**Discussion:**

The certainty of the evidence is low, and a causal effect of microdosing cannot be inferred. Future research should prioritize well-powered randomized controlled trials with standardized and substance-specific dosing protocols to determine causal relationships between microdosing MM or truffles and health outcomes. Mandatory reporting of adverse events and funding sources should be implemented to build the safety profile of microdosing MM and truffles and ensure transparency for potential conflicts of interest.

**Sytematic review registration:**

https://www.crd.york.ac.uk/prospero/, identifier CRD42023428107.

## 1 Introduction

Psilocybin, a naturally occurring alkaloid psychedelic compound found in certain species of mushrooms (1) and truffles (2), has emerged as an area of scientific investigation owing to its potential therapeutic effects on mental health and well-being. Several recent systematic reviews have demonstrated psilocybin improves mental health outcomes (3–7), domains of cognitive function, including flexibility (8) and executive functioning (9), social functioning (5, 8), emotional regulation (5), creativity (8), and substance use (10). However, most of these reviews included interventions testing full doses of psilocybin combined with psychotherapy sessions (4–9), when much of the public and scientific attention has increasingly turned to psilocybin microdosing in the community and largely outside regulated clinical and research settings (11).

Microdosing involves the repeated administration of sub-perceptual doses of psychedelic drugs, most often from psilocybin-containing magic mushrooms (MM) or truffles (12) and lysergic acid diethylamide (LSD) (11, 13) and may be promising for treating mental health conditions (13, 14). A systematic review has suggested that microdosing may enhance mental well-being, including mood elevation, improved focus, and better daily functioning, but these findings were from studies that included multiple psychedelic substances (15). Likewise, Ona et al., 2020 identified inconsistent effects on mood, creativity, and energy, but its reliance on mixed samples of psychedelic and non-psychedelic agents limits substance-specific conclusions (16). In contrast, a meta-analysis by Pinhas and colleagues examined cognitive outcomes and found a significant decrease in cognitive control when participants microdosed with LSD or psilocybin (17). Notably, their subgroup analysis restricted to psilocybin alone revealed no measurable impact on cognitive function, suggesting that the specific psychedelic substance may critically influence observed effects. Taken together, the extent to which benefits can be attributed to microdosing MM or truffles remains unclear, necessitating substance specific investigation (11).

The practice of administering psychedelics at low doses is not new, and distinguishing its historical therapeutic use from its current wellness-oriented form is important for interpreting this literature. From the 1950s, lower doses of LSD and psilocybin were used within supervised psychotherapy under a model later termed “psycholytic therapy,” in which doses were intended to produce mild but clearly perceptible effects to facilitate the therapeutic process (18). This differs fundamentally from the contemporary concept of microdosing, popularized through lay sources in the 2010s (12), in which repeated sub-perceptual doses are self-administered, typically without clinical supervision and for wellness or performance rather than for treatment of a diagnosed condition (18, 19). Recognizing this distinction matters because efficacy signals from supervised, perceptible-dose therapy cannot be assumed to transfer to unsupervised, sub-perceptual wellness use.

A parallel body of non-human research has examined microdosing regimens in animal models, and its findings temper expectations drawn from naturalistic human reports. A 2025 narrative review of 12 studies administering LSD, psilocybin, or DMT to rats, mice, and zebrafish concluded that microdosing produced little change in anxiety- or depression-related behaviors and was generally well tolerated, while cautioning that the preclinical evidence base is small and methodologically heterogeneous (20). Individual studies are mixed: chronic, intermittent-low dose DMT produced antidepressant-like and enhanced fear-extinction effects in rodents (21), whereas psilocybin and LSD produced in a rodent model (22). While animal models do permit blinding, randomization, and control of expectancy that naturalistic human studies cannot, the generally modest and inconsistent preclinical signal is informative and may suggest that some of the benefit reported in uncontrolled human studies may reflect expectancy rather than a direct pharmacological effect of sub-perceptual dosing.

The scientific literature on microdosing is characterized by substantial variability in study design, dosing regimens, psilocybin preparations, and outcome measures, as well as a reliance on self-report and observational methodologies (13, 15–17, 23). Much of the literature has noted the potential influence of expectancy effects and placebo responses on reported outcomes from psychedelics combined (11, 15–17), and limited data exist regarding side effects, adverse effects, safety, and long-term consequences of psilocybin MM and truffle microdosing (11, 13, 14, 16). Full-dosed psilocybin interventions have been found to be safe with minimal adverse effects reported (4, 6, 7, 10), but due to the potentially chronic, long-term nature of microdosing, further research is warranted (11, 13, 14, 16).

While some systematic reviews have examined microdosing effects across multiple psychedelics, including psilocybin, LSD, and synthetic derivatives (11, 15–17), this mixed approach restricts the ability to draw conclusions specific to psilocybin MM and truffles which are being microdosed in the community setting (11, 13). Despite growing public and scientific interest, systematic reviews describing the reported effects, dosing parameters, safety profile (23), and sources of bias for psilocybin microdosing remain limited (15–17). Furthermore, the lack of standardized definitions and dosing schedules for psilocybin microdoses creates challenges in comparing and evaluating outcomes. Given these methodological challenges, a rigorous synthesis of the available evidence is needed to clarify what is currently known and identify knowledge gaps related to microdosing psilocybin MM or truffles.

The purpose of this systematic review was to examine and synthesize adult psilocybin MM or truffle microdosing, including reported health effects, perceived benefits, motivations or indications for use, dosing protocols and preparations, side effects and adverse events, and differences by sex and age. Funding sources and competing interests were also explored.

## 2 Methods

This systematic review followed the Preferred Reporting Items for Systematic Reviews and Meta-Analyses (PRISMA) (24) and was registered with the International Prospective Register of Systematic Reviews (https://www.crd.york.ac.uk/prospero/), CRD42023428107.

### 2.1 Data sources and searches

A literature search was conducted in March and April 2025, and updated in June 2026, using CINAHL, OVID, ProQuest, PsycINFO, PubMed, and Scopus databases using the same search string, ((psilocybin*) OR (psilocin) OR ("magic mushroom*")) AND (micro*) OR ("sub-acute") OR ("low dos*"). No constraints were used on publication dates and only studies published in English or Spanish peer-reviewed journals were included. Searches of the databases were conducted by a reference librarian, and after compilation of study titles, they were uploaded into Covidence software (25). Duplicate titles were then removed and confirmed by two reviewers (AM, JP). Reference lists of included studies and other relevant systematic reviews were manually examined to identify any additional studies for potential inclusion.

### 2.2 Study screening and selection

The screening process involved an examination of the study title and abstract by three reviewers (BH, AM, JP) for inclusion based on the PICO (population - adults, intervention - microdosing with psilocybin MM or truffles, comparator - placebo or none, outcome - any health related). Inclusion criteria included: (1) analyses of findings in adults aged 16 years and older; (2) quantitative or qualitative study designs; and (3) examined microdosing with psilocybin MM or truffles. Preclinical studies without adult human primary data were excluded. Microdosing was operationalized as a microdose if study authors explicitly stated participants were microdosing or if the administered dose met predefined quantitative thresholds, defined as dried or fresh psilocybin MM dosages of less than 0.5 grams (11, 13) or fresh psilocybin truffles of less than 1 gram. The truffle dosage of 1 gram was chosen as a cut off because a microdose is approximately 1/10^th^ of a full dose (12) which was identified in the lay literature as 10 g (26, 27). Of note, a peer-reviewed source for the actual dosages for truffle microdosing or full dosing was not identified through searches; therefore, lay sources were used. Studies involving synthetic psilocybin, psilocybin isolates, or testing other treatments combined with microdosing MM or truffles were excluded.

### 2.3 Data extraction

Data extracted from each study included: (1) aim or purpose of study; (2) study design; (3) participant characteristics (e.g., sex, age, race/ethnicity, income, education, employment); (4) health related outcome and measures; (5) study findings for health outcomes or perceived benefits with differences by sex and age; (6) motivations for personal use or indications for research; (7) microdosing practices (psilocybin dosage, dosing schedule, preparations, strains); (8) side effects and adverse events; (9) funding sources; and (10) conflicts of interest. Side effects and adverse events were differentiated during extractions, as the two concepts are distinct. Side effects encompass a spectrum of outcomes ranging from mild to serious (28), whereas adverse events refer specifically to unexpected and severe occurrences such as hospitalization, life-threatening reactions, and death (29). Four reviewers (BH, EL, AM, JP) independently extracted data with two reviewers assigned to each article. Results were compared between reviewers and disagreements were resolved through group discussion.

### 2.4 Quality assessment

Study quality for the qualitative studies was assessed using Joanna Briggs Institute (JBI) Critical Appraisal Tool for Qualitative Research (30). For the quantitative study designs, Cochrane RoB 2 (31) was used for randomized trials, ROBINS-I (32) for non-randomized, and ROBINS-E (33) for observational studies. Four reviewers (BH, EL, AM, JP) conducted the quality assessments independently, two reviewers were assigned to each article, and disagreements were addressed through discussion.

### 2.5 Data synthesis and analysis

To synthesize the study characteristics and findings, the Cochrane Data Synthesis and Analysis Narrative Synthesis Guidelines (34) were followed with data synthesized to include: (1) participant characteristics (sex, age, race/ethnicity, income, education, employment); 2) description of findings for health effects; 3) motivations or indications for use and/or research; 4) psilocybin microdosing practices; 5) side effects; 6) adverse events; 7) funding sources; 8) conflicts of interest; 9) strengths; 10) limitations; 11) quality assessment. The analysis involved compiling a comprehensive narrative synthesis of the findings from studies included and examining the patterns and relationships identified in the study data.

## 3 Results

**Figure 1:**
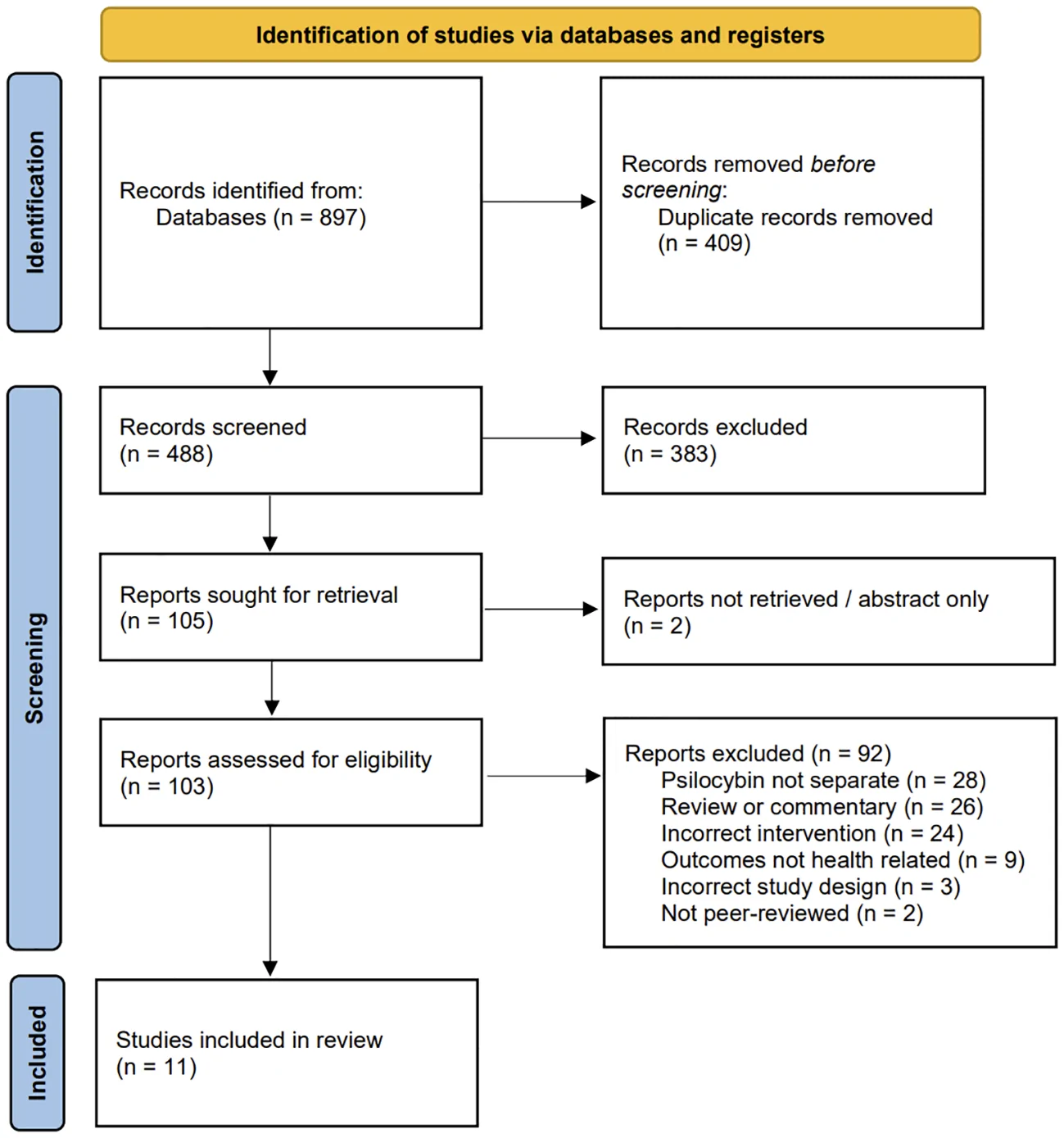
PRISMA 2020 flow diagram for new systematic reviews which included searches of databases and registers only. Adapted from Page et al., (2021), CC BY 4.0, doi: 10.1136/bmj.n71 (58).

The PRISMA flow diagram (**Figure 1**) outlines the screening and selection procedure for this systematic review. A comprehensive database search conducted by a reference librarian yielded 897 references, which were imported into Covidence, leaving 488 references after duplicates were removed. After screening titles and abstracts, 105 reports were sought for retrieval and 103 articles underwent a comprehensive full-text review, and 11 articles (10 in English and 1 in Spanish) met the inclusion criteria. We used ChatGPT (35) to translate the Spanish article into English and then verified the translation with a bilingual Spanish-certified medical translator.

### 3.1 Study characteristics

Detailed information for each study is presented in **Table 1**. Included studies were published between 2018 (36) and 2024 (37, 38). Four out of the eleven studies (36.4%) were conducted in the United States (39–42). The remaining seven studies (63.6%) were conducted in countries outside of the United States, including two (18.2%) international surveys (43, 44), two studies (18.2%) conducted in Argentina (38, 45), two (18.2%) in the Netherlands (36, 46), and one study (9.1%) conducted in Slovenia (37).

**Table 1:** Study findings.

Author (year) setting	Sample descriptives study design	Psilocybin type (%) study groups	Findings
Quantitative studies			
Cavanna (45) (2022) Argentina	*n* = 34 Male = 23 (67.6%) Female = 11 (32.4%) *Mean age* = 31.3 years Double-blind, placebo-controlled study	MM (100.0) IG vs. CG CG = Suillus granulatus	• Significantly decreased theta waves on brain activity (electroencephalogram [EEG]) with closed eyes in IG vs. CG (p < 0.05) • No significant effect on cognition and perception (Attentional Blink [AB], Go/No-Go, Trail Making Test [TMT], Backward Masking [BM], and Binocular Rivalry [BR], Stroop Task; all p > 0.05 after correction for multiple comparisons) for IG vs. CG • No significant differences in creativity ((Remote Associates Test [RAT]), (Alternative Uses Tasks [AUT]), (Wallach-Kogan Test [WK]); all p > 0.05), self-reported measures of mental health and wellbeing (State–Trait Anxiety Inventory [STAI], Positive and Negative Affect Schedule [PANAS], Perceived Stress Scale [PSS], Psychological Well-Being Scale [BIEPS]; all p > 0.05), or physical activity (Fitbit) participation between IG vs. CG (p > 0.05)
Lea (43) (2020) International	*n* = 242 **Male = 169 (69.8%) **Female = 73 (30.2%) *Mean age* = 33.6 years Cross-sectional	MM (93.8) Truffles (6.6)	• Perceived benefits participants usually experienced on more than 50% of days they microdosed: Less depressed than usual (62.4%), happier than usual (62.4%), less stressed than usual (57.9%), feels more connected to nature and other living things (55.8%), higher level focus/concentration (53.7%), less anxious than usual (53.3%), enhanced senses (52.9%), less irritable than usual (52.1%), feels more connected to other people (52.1%), more empathetic than usual (50.8%), more stamina/energy than usual (38.4%); self-report using Lea, Amada & Jungaberle (2019) taxonomy
Marschall (46) (2022) Netherlands	*n* = 52 (S1/S3); 44 (S2/S4) Male = 23 (44.2%) (S1/S3); 23 (52.3%) (S2/S4) Female = 29 (55.8%) (S1/S3); 21 (47.7%) (S2/S4) *Mean age* = 29.75 years (S1/S3); 30.2 years (S2/S4) Double-blind, placebo-controlled within subject crossover study	Truffles (100.0) IG vs. CG CG: Dried non-psychoactive mushrooms and seeds	• No significant difference in anxiety, depression, stress (Shortened Depression Anxiety Stress Scale [DASS-21]; Lovibond and Lovibond, 1995) between IG vs. CG (p>0.05) across S2 and S4 • No significant differences in multidimensional awareness (Multidimensional Assessment of Interoceptive Awareness Scale [MAIA]; Mehling et al., 2012) subscales or emotional processing (Emotional go/no-go task; Erickson et al., 2005; Schulz et al., 2007) between IG vs. CG (p>0.05) across S1 and S3 • In S1, CG scored higher on emotional awareness (MAIA subscale; p=0.024) and self-regulation (MAIA subscale; p=0.02) vs. IG but not in S3 (p>0.05). • No main effect of condition and no Condition × Emotion interaction on the Emotional Go/No-Go Task (p>0.05) • No significant interaction by sex for anxiety, depression, emotional processing, interoceptive awareness, or stress (p>0.05) IG and CG were administered their respective dose before arrival to the session where data collection occurred
Petranker (44) (2022) International	*n* = 2832 Male = 2011 (71.0%) Female = 821 (29.0%) *Median age* = 26.0 years Cross-sectional	MM (100.0)	• Perceived benefits ranked from highest to lowest: Enhanced mood and decrease depressive symptoms (56.8%), enhanced creativity and curiosity, enhanced empathy, sociability or communication (41.0%), enhanced energy and alertness (39.3%), reduced stress (38.2%), enhanced productivity, motivation or confidence (33.8%), reduced social anxiety (33.2%), enhanced sight, hearing, smell, athletic performance or sleep (31.2%), enhanced focus (30.0%), enhanced mental clarity and memory (29.2%), reduced substance dependence symptoms (14.7%); self-report using Anderson et al., 2019a taxonomy) • 5.4% experienced no effect at all
Prochazkova (36) (2018) Netherlands	*n* = 38 Male = 23 (60.5%) Female = 15 (39.5%) *Mean age* = 31.1 years Non-blind, quasi-experimental open-label natural setting study	Truffles (100.0) IG only	• Significant improvement in convergent thinking (Picture Concept Task [PCT]) for IG between T1 and T2 (p=0.017) • Significant improvement in divergent thinking (Alternate Uses Tasks [AUT]) for IG between T1 and T2 (p=0.009), with significant improvement in divergent thinking subcomponents fluency (AUT; p =0.024), flexibility (AUT; p = 0.018), and originality (AUT; p = 0.002) for IG between T1 and T2. • No significant improvement in divergent thinking subcomponent elaboration scores for the IG (p > 0.05) between T1 and T2 • No significant improvement in fluid intelligence (Raven’s Progressive Matrices [RPM]) for the IG (p > 0.05) between T1 and T2
Qualitative studies			
Kinderlehrer (39) (2023) USA	*n* = 1 Male = 1 (100%) *Mean age =* 70.0 years Case study	MM (100.0)	• Improved mood within 2 days • Consistently feeling well within 2 weeks • Anxiety and depression in remission at 2 years
Kovacevich (40) (2023) *USA	*n* = 1 Female = 1 (100%) *Mean age* = 22.0 years Case study	MM (100.0)	• Improved PCR-confirmed COVID-19 induced anosmia (not measured) at 1 day post 3 consecutive days of MM • Anosmia scored 15 (University of Pennsylvania Smell Identification Test, UPSIT) at 2-months; this score indicates anosmia; however, reported continued subjective improvement • Improved anosmia persisted (not measured) at 8 months
Lyes (41) (2023) USA	*n* = 3 Male = 1 (33.3%) Female = 2 (66.7%) *Mean age* = 48.6 years Case study	MM (100.0)	• Improved neuropathic pain (self-reported pain scale 0-10) (3/3) • Improved flexibility (1/1), functional ability (2/2), muscle spasms (1/1), neurogenic bowel symptoms (1/1) and quality of life (1/1) participants
Lyons (42) (2022) USA	*n* = 1 Male = 1 (100%) *Mean age* = 43.0 years Case study	MM (100.0)	• Improved depression from baseline score of 27 (Hamilton Depression Rating Scale) to 20 at 1-week, 11 at 6-months, and 7 at 2-years (classified in remission) • Improved anxiety, appetite, delusions of guilt, and libido at 1-week • Improved anhedonia and suicidal ideation at 6-months • Normal functioning at 2-years
Oblak (37) (2024) Slovenia	*n =* 13 Male = 4 (30.8%) Female = 9 (69.2%) *Mean age* = 27.2 years Mixed-methods study	MM (100.0)	• Improved flexible cognition and increased creativity, mindfulness, and salience of mundane events • Some participants experienced decreased stable cognition, poor inhibitory control, tangential stream of consciousness • Increased salience of implicit social dynamics, loosening of inhibitions, new and sometimes ego-dystonic affective states, adopted social roles (acted or felt in ways they found inconsistent with their normal identity) • Developed symptoms of psychosis
Zarankin (38) (2024) Argentina	*n =* 1 Non-binary = 1 (100%) *Mean age* = 19.0 years Case study	MM (100.0)	• At 0.2 g, mild mood improvements (not measured), better work performance and concentration; however, had persistent fatigue and continued sense of existential emptiness and passive thoughts of death • At 0.37 g, improvements in mood (not measured) and energy; however, libido slightly decreased • At one month, improved autonomy, communication, energy, frustration tolerance, household collaboration, mood, motivation, self-care, and social interaction (peer, family, authority figures); Quetiapine tapering started • At 6 weeks, fatigue and low energy returned, psilocybin dose lowered to 0.2 g and quetiapine tapering continued • At 2 months, psilocybin was adjusted to 5 days/week and quetiapine was discontinued; communication, creativity, decreased irritability, emotional regulation, energy, mood, and sociability improved; death ideation remitted; parents reported better emotional closeness and communication • Sertraline gradually tapered with no return of symptoms (no time frame provided) • Psilocybin reduced to 0.1 g and stopped one month later; depression score reduced from 9 to 0 (Hamilton Depression Rating Scale)

MM, magic mushrooms; IG, intervention group; CG, control group; MD, microdosing; S, session; *where IRB approval received; **, demographic information only available for the any psilocybin group and not psilocybin only group. Unless specified, outcomes were not measured.

Five studies were quantitative (45.5%; 5/11), including two (40%) cross-sectional studies (43, 44), one (20%) double-blind, placebo-controlled study (45), one (20%) double-blind, placebo-controlled within-subject crossover study (46), and one (20%) non-blinded, quasi-experimental open-label natural setting study (36). Six studies (54.5%) used qualitative designs; five (83.3%) were case studies or case reports (38–42), and one (16.7%) employed a mixed-methods qualitative approach (37).

Across the 11 included studies, a total of 3,262 participants were represented. Sample sizes ranged from a single participant (38–40, 42) to 2,832 (44), with five studies reporting fewer than five participants (38–42). Overall, the pooled sample included 2,279 males (69.9%), 982 females (30.1%), and 1 participant (0.03%) who identified as non-binary. All studies reported participant age, with mean or median ages ranging from 19 (38) to 70 years (39).

Race, ethnicity, and socioeconomic status were not consistently reported across the studies. In one multi-participant study (43) (Lea et al., 2020), information on education level and employment status was provided, indicating that 277 participants (52.8%) had completed a university degree; 259 (49.3%) were employed full-time, 81 (15.4%) part-time, 91 (17.3%) identified as students, and 94 (17.9%) selected “other” employment status. Additionally, one case study reported a single male participant who identified as White/Caucasian, was a high school graduate, employed as a real estate agent, and reported stable socioeconomic status (42). The remaining studies did not report race, ethnicity, education, or socioeconomic information.

### 3.2 Effects of microdosing on health-related outcomes

Reported effects and benefits of microdosing on health-related outcomes are presented in **Table 1**. Across the 11 studies, eight studies (72.7%) reported at least one positive effect or benefit of microdosing on a health outcome (36, 38–44). One study (9.1%) reported both negative and positive effects (37) and two studies (18.2%) reported no significant improvements with psilocybin microdosing on most health outcomes (45, 46). Better scores in the control groups compared to the intervention groups (45, 46) were identified in some outcomes.

The outcomes reported across studies were not fully aligned with the motivations for microdosing (**Table 2**) or conceptual indications for research (**Table 3**), and several studies examined additional outcomes that were not included in both tables. The findings are presented on all specified outcomes, which have been categorized into the following seven domains, well-being, mental health, cognition, physical health, social relationships, creativity, enhanced senses, and additional outcomes.

**Table 2:** Microdoser motivations for use, *n* (%).

Author (year)	Mental health^a^	Well-being^b^	Cognition^c^	Curiosity^d^	Avoid bad habits^e^	Physical health^f^	Enhance creativity	Improve relationships	Chronic pain	Post-COVID 19 induced anosmia	Lyme disease^g^
Kinderlehrer (39) (2023)											1 (100.0)
Kovacevich (40) 2023										1 (100.0)	
Lea (43) (2020)	111 (45.9)	67 (27.7)	40 (16.5)	15 (6.2)	3 (1.2)	6 (2.5)					
Lyes (41) (2023)									3 (100.0)		
Lyons (42) (2022)	1 (100.0)										
Oblak* (37) (2024)	3 (23.1)	3 (23.1)	7 (53.8)			1 (7.7)					
Petranker* (44) (2022)	210 (10.0)	606 (29.0)	81 (3.9)	714 (34.1)	60 (2.9)		288 (13.8)	133 (6.4)			
Zarankin (38) (2024)	1 (100.0)										
Total participants *N* = 2,355	326 (13.8)	676 (28.7)	128 (5.4)	729 (31.0)	63 (2.7)	7 (0.3)	288 (12.2)	133 (5.6)	3 (0.1)	1 (0.04)	1 (0.04)
*N* (%) of studies	5 (62.5)	3 (37.5)	3 (37.5)	2 (25.0)	2 (25.0)	2 (25.0)	1 (12.5)	1 (12.5)	1 (12.5)	1 (12.5)	1 (12.5)

Mental health^a^ includes mental health, anxiety, depression, attention deficit and hyperactivity disorder; Well-being^b^ includes well-being, mood, life satisfaction, self-knowledge; Cognition^c^ includes cognitive enhancement, productivity; Curiosity^d^ includes curiosity, boredom, unknown reasons; Avoid bad habits^e^ includes bad habits, substance use, unhealthy behaviors; Physical health^f^ includes physical health, somatic illness; Lyme disease includes neuropsychiatric Lyme disease; Oblak* totals more than 100% because participants could choose more than one; Petranker* only provided indications for 73.9% (2,092) of the total sample.

**Table 3:** Conceptual indications for research.

Author (year)	Cognition	Creativity (divergent and convergent thinking)	Stress	Anxiety	Depression	Subjective experience	Emotional processing	Interoceptive awareness	Brain activity	Perception	Behavior
Cavanna (45) (2022)	X	X				X			X	X	X
Marschall (46) (2022)			X	X	X		X	X			
Prochazkova (36) (2018)	X	X									
*N* (%) studies	2 (66.7)	2 (66.7)	1 (33.3)	1 (33.3)	1 (33.3)	1 (33.3)	1 (33.3)	1 (33.3)	1 (33.3)	1 (33.3)	1 (33.3)

#### 3.2.1 Well-being

Nine of the 11 studies (81.8%) examined well-being related outcomes (37–39, 41–46). Of those nine, six (66.7%) reported improvements/benefits in well-being related outcomes (38, 39, 41–44). Only one of the studies assessed the concept of well-being (45) and did not demonstrate any significant change. Studies reported improvements in mood or enhanced mood (38, 39, 44), feeling happier (43), feeling well after dosing (39), irritability (38, 43), frustration and tolerance (38), energy levels and alertness (38, 43, 44), sleep (44), and quality of life (41). One study reported a decrease in delusions of guilt (42). Stress improved in two studies (43, 44) but there was no change in stress in two other studies (45, 46). One study demonstrated mixed findings in well-being outcomes (37) with an improvement in mindfulness but also demonstrated salience of mundane events.

#### 3.2.2 Mental health

Eight of the eleven studies (72.7%) provided findings for at least one mental health-related outcome (37–39, 42–46) with 62.5% demonstrating improvements or benefits, two studies did not demonstrate any changes (45, 46), and one reported negative mental health findings (37). Six studies examined depression-related outcomes (38, 39, 42–44, 46), with five (83.3%) reporting improvement in symptoms (38, 39, 42–44) and one reporting no significant change (46). Similarly, five studies examined anxiety-related symptoms (39, 42, 43, 45, 46) with three (60%) reporting improvement in symptoms (39, 42, 43) and two reporting no significant change (45, 46). Reductions in suicidal or death ideation (38, 42) and anhedonia (42) were reported. Changes in libido were reported, one study demonstrated positive changes (42) and one reporting a slight decrease (38). One study reported participants experiencing psychosis (37).

#### 3.2.3 Cognition

Seven studies (63.6%) examined cognition-related outcomes (36–38, 43–46) with four studies (57.1%) reporting positive improvement (36, 38, 43, 44), two studies reporting no significant findings (45, 46) and one reported mixed findings (37). Specifically, with positive symptoms, studies reported higher focus and concentration (38, 43, 44), enhanced productivity and better work performance (38, 44), and improvements in autonomy, motivation, and confidence (38, 44), convergent and divergent thinking (36), emotional regulation (38) and mental clarity and memory (44). No significant improvements were demonstrated in cognition, perception (45), emotional processing, emotional awareness, interoceptive awareness and self-regulation (46). The control group demonstrated better scores on emotional awareness and self-regulation at one time point (46). One study reported contradictory findings with participants reporting improved flexible cognition but they also experienced decreased stable cognition, including poor inhibitory control and a tangential stream of consciousness (37).

#### 3.2.4 Physical health

Four of the 11 studies (36.4%) examined physical health-related outcomes (38, 41, 44, 45). Among these, three (75.0%) reported positive physical health outcomes (38, 41, 44) and one study reported no significant change (45). Studies reported improvements in flexibility, functional ability, muscle spasms, neurogenic bowel symptoms (41), athletic performance (44), and self-care (38). One study did not demonstrate significant improvements in objectively-measured physical activity (45).

#### 3.2.5 Social relationships

Four of the 11 studies (36.4%) examined social relationship-related outcomes (37, 38, 43, 44) with three (75.0%) reporting positive outcomes and one study reporting mixed findings (37). Participants reported feeling more connected or emotionally close (38, 43) and empathy (43, 44). Improvements in sociability and/or social anxiety (38, 44) and communication and/or collaboration (38, 44) were reported. Participants in one study also demonstrated negative changes in social dynamics that included loosening of inhibitions, ego-dystonic affective states, and adopted social roles that were inconsistent with their usual identity (37).

#### 3.2.6 Creativity

Five studies (45.5%) examined creativity outcomes (36–38, 44, 45). Four of the studies (80.0%) reported positive creativity-related outcomes (36–38, 44), while one study reported no significant change (45).

#### 3.2.7 Enhanced senses

Enhanced senses were included in three (27.3%) of the 11 studies. All (100%) reported positive improvements, including improved post-COVID-19 induced anosmia (40), enhanced sight, hearing, and smell (44), and enhanced senses overall (43).

#### 3.2.8 Additional outcomes

Substance dependence symptoms (44), chronic pain (41), and curiosity (44) demonstrated improvements in one study each.

### 3.3 Motivations for microdosing

As shown in **Table 2**, motivations for microdosing were reported across multiple domains. Curiosity (includes curiosity, boredom, and unknown reasons) was the most frequently endorsed motivation, reported by 729 participants (31.0%) from two studies (43, 44). Well-being related motivations (includes mood, life satisfaction, and self-knowledge) were reported by 676 participants (28.7%) across three studies (37, 43, 44). Mental health motivations (includes anxiety, depression, and attention deficit/hyperactivity disorder) were reported by 326 participants (13.8%) across five studies (37, 38, 42–44). Cognition (includes cognitive enhancement and productivity) were reported by 128 participants (5.4%) across three studies (37, 43, 44). Avoid bad habits (includes bad habits, substance use, unhealthy behaviors) was reported by 63 participants (2.7%) in two studies (43, 44). Physical health–related motivations were infrequently reported (*n* = 7; 0.3%) and appeared in two studies (37, 43). Additional motivations included creativity enhancement (*n* = 288; 12.2%) (44), relationship improvement (*n* = 133; 5.6%) (44), chronic pain reduction (*n* = 3; 0.1%) (41), and symptom relief related to post COVID-19 induced anosmia (*n* = 1; 0.04%) (40) and Lyme disease (*n* = 1; 0.04%) (39).

### 3.4 Conceptual indications for research

Conceptual indications for research were described in three studies (**Table 3**). Of those studies, two studies (66.7%) examined cognition and creativity, including divergent and convergent thinking (36, 45). One study (33.3%) focused on stress, anxiety, depression, emotional processing, and interoceptive awareness (46), while Cavanna et al. (33.3%) also assessed subjective experience, brain activity, perception, and behavior (45).

### 3.5 Dosing protocols and preparations

The psilocybin preparation, dose, and microdosing schedule are shown in **Table 4**. Participants in three out of the 11 studies (27.3%) microdosed truffles in dried or encapsulated form (36, 43, 46) and the remaining studies (8/11; 72.7%) used magic mushrooms in various forms, including dried, ground, encapsulated, in chocolate, or fresh and cut up (37–42, 44, 45). Reported doses ranged from 0.05 (38, 41) to 1.0 gram (41). Microdosing frequency and duration varied widely, ranging from a single dose (36) to daily administration (37, 41, 43), with one study not specifying frequency or duration (44).

**Table 4:** Dosing practices.

Author (year)	Psilocybin (*species*) preparation	Dosage	Schedule
Cavanna (45) (2022)	MM (*Psilocybe cubensis*) Dried, ground & encapsulated	0.5 g	• 2 doses for 1 week
Kinderlehrer (39) (2023)	MM *(Not provided)* Dried whole	0.1 to 0.125 g	• 0.1 g for 2 weeks, increase to 0.125 g • 3 times per week for ≥ 2 years
Kovacevich (40) (2023)	MM *(Not provided)* Dried	0.1 g	• 3 consecutive nights
Lea (43) (2020)	MM & Truffles (*Not provided*) Dried & cut up Dried & ground Pre-ground Encapsulated Fresh & cut up	<0.1 to >0.5 g Unknown dose – 4.5%	• Daily • Every other day • 1 day on, 2 days off (most common) • 5 days on, 2 days off • Every 4 days (1–2 times/week) • Once a week • Once a fortnight • Flexible schedule, as needed • Something else
Lyes (41) (2023)	MM (*Not provided*) (1, 2) Dried & ground (3) Dried & ground in chocolate bar	(1) 0.25 g; reduced 0.05 g (2) 0.5 g; 0.75 to 1.0 g (3) 1.0 g	• (1) ~Daily for 6 months • (2) Daily for 7–10 days, 2–3 days off, for > 1 year; higher dose range occasionally • (3) Once every 2 months
Lyons (42) (2022)	MM (*Psilocybe cubensis*) Dried & ground, encapsulated	0.1 to 0.2 g	• 1 dose on days 1, 4, & 7 • 3 doses per week for 8 weeks, then 4-week break, repeated for 3 years
Marschall (46) (2022)	Truffles (*Psilocybe galindoi*) Dried, encapsulated	0.7 g	• Between 5–7 doses per 3 weeks with at least 1 day between doses, dose taken within 45 minutes to 2.5 hours before each lab session
Oblak (37) (2024)	MM (*Not provided*) Dried Chocolate	0.1 to 0.3 g	• Frequency: variable (once weekly, Fadiman [Q 3 days], up to 6 days/week) • Duration: not standardized; 8-day microdosing observation period (4 dosing days, 4 non-dosing days off)
Petranker (44) (2022)	MM (*Not provided*) Not specified	<0.2 g	• Not specified
Prochazkova (36) (2018)	Truffles (*Not provided*) Dried	0.22 to 0.44 g	• Once
Zarankin (38) (2024)	MM (*Not provided*) Dried & ground	0.05 to 0.37 g	• Every other day for three months • Five days per week for one month (for remainder of seven month study duration)

MM, magic mushrooms.

### 3.6 Side effects and adverse events

Four of the 11 studies (36.4%) reported one or more side effects associated with psilocybin microdosing (37, 41, 42, 44). One study (9.1%) explicitly reported no side effects (38). Six studies (54.5%) did not report side effects (36, 39, 40, 43, 45, 46).

Among studies reporting side effects, gastrointestinal and appetite-related symptoms were reported in four studies (36.3%), including nausea (42), stomach pain (44), flare up of Crohn’s disease (37), and diminished or lost appetite (41, 44). Two studies (18.2%) reported anxiety-related symptoms (37, 44), one of which noted suicidal ideation at higher doses (37), though these symptoms were not reported as adverse events. Cognitive or perceptual effects were reported in three studies (27.3%), including disorientation (41), impaired focus (44), mental confusion (44), dissociation (44), and sensory distortions (37).

Seven studies (63.6%) did not report adverse events related to psilocybin microdosing (36, 39, 40, 42, 44–46). One study (9.1%) explicitly reported no adverse events (38), one study (9.1%) reported adverse events did occur with symptoms of psychosis, sensory distortion, and suicidal ideation at higher doses (37), and one study (9.1%) did not disaggregate adverse events for psilocybin-only microdosing (43). Findings on side effects and adverse events are presented in **Table 5**.

**Table 5:** Side effect and adverse event reporting.

Author (year)	Side effects	Adverse events	Describe
Cavanna (45) 2022	Not reported	Not reported	
Kinderlehrer (39) 2023	Not reported	Not reported	
Kovacevich (40) 2023	Not reported	Not reported	
Lea (43) 2020	Not reported	Reported but not for psilocybin-only microdosing	
Lyes (41) 2023	Reported	Reported none occurred	Minimal cognitive or somatic adverse effects; diminished appetite; disorientation; gait stability
Lyons (42) 2022	Reported	Not reported	Mild nausea after first two doses
Marschall (46) 2022	Not reported	Not reported	
Oblak (37) 2024	Reported	Reported	Anxiety (3 days of intense physiological symptoms of anxiety at the end of the microdosing period)/panic attack; symptoms resembling psychosis at higher doses: sensory distortions, hyper-reflexivity; suicidal ideation; flare up of Crohn’s disease
Petranker (44) 2022	Reported	Not reported	Physiological discomfort (Stomach pain, headache, sleep problems, loss of appetite); impaired/reduced focus; impaired energy (restlessness and/or fatigue); mental confusion, memory problems, or racing thoughts; increased anxiety, including social anxiety; dissociation or rumination; negative mood, irritability or instability; legal consequences/illegality; unpredictable effects and/or negative drug interactions; social problems; substance dependence symptoms and hard comedown
Prochazkova (36) 2018	Not reported	Not reported	
Zarankin (38) 2024	Reported none occurred	Reported none occurred	

### 3.7 Funding sources and competing interests

Funding sources and conflicts of interest reporting were summarized in **Table 6**. Five of the 11 studies (45.5%) reported some form of funding support, including four with government funding (36, 40, 43, 45), one received support from private, non-profit organizations (36), and one received industry-related material support in the form of psilocybin truffles (46). Two studies (18.2%) explicitly reported no financial support (42, 44), and four studies (36.4%) did not report funding sources (37–39, 41). Eight studies (72.7%) declared no conflicts of interest (36–39, 42, 43, 45, 46), while three (27.3%) reported conflicts (40, 41, 44).

**Table 6:** Significant findings by funding source.

Author (year)	Funding	Reported conflicts of interest	Any significant or symptom(s) improvement	Industry-related funding source
Cavanna (45) 2022	Government	Declared no conflicts	–	No
Kinderlehrer (39) 2023	Not reported	Declared no conflicts	+	Unknown
Kovacevich (40) 2023	Government	Yes	+	No
Lea (43) 2020	Government	Declared no conflicts	+	No
Lyes (41) 2023	Not reported	Yes	+	Unknown
Lyons (42) 2022	No financial support received	Declared no conflicts	+	No
Marschall (46) 2022	No financial support received MagicTruffles.com provided the psilocybin-containing truffles	Declared no conflicts	–	Yes
Oblak (37) 2024	Not reported	Declared no conflicts	+	Unknown
Petranker (44) 2022	No financial support received	Yes	+	No
Prochazkova (36) 2018	Psychedelic Society of The Netherlands provided space for experiments (Private, non-profit) Advanced Grant of the European Research Council (Government) MICR (Ministry of the Interior of the Czech Republic) Self-supplied	Declared no conflicts	+	No
Zarankin (38) 2024	Not reported	Declared no conflicts	+	Unknown

### 3.8 Study quality assessment

Quality assessments for each study are presented in **Table 7**. Among the five quantitative studies (36, 43–46), both of the two randomized controlled trials (45, 46) were rated as having some concerns overall, primarily due to issues in deviations from intended interventions and missing outcome data. Of the two observational studies, one was rated with some concerns (43), while the other was rated as very high risk of bias (44), driven by high risk in multiple domains, including confounding, missing data, and exposure measurements. The quasi-experimental study (36) was rated as having serious risk of bias, with most significant concerns related to confounding and selection bias.

**Table 7:** Quality assessment.

Author (year)	Domain 1	Domain 2			Domain 3				Domain 4			Domain 5				Domain 6		Domain 7			Overall		
*Quantitative*																							
*ROB-2*																							
Cavanna (45) (2022)	Low		Some Concerns		Low				Low	Low						NA		NA			Some Concerns		
Marschall (46) (2022)	Low		Some Concerns		Some Concerns				Low	Low						NA		NA			Some Concerns		
ROBINS-E																							
Lea (43) (2020)	Low		Some Concerns		Low				Low	Some Concerns					Low			Low			Some Concerns		
Petranker (44) (2022)	High Risk		Low		Low				Low	High Risk					High Risk			Low			Very High Risk		
ROBINS-I																							
Prochazkova (36) (2018)	Serious		Low		Low				Low	Moderate					Serious			Low			Serious		
Qualitative																							
Authors Year	Q1	Q2		Q3			Q4	Q5			Q6		Q7	Q8			Q9		Q10			Overall	
JBI Critical Appraisal Checklist for Case Reports																							
Kinderlehrer (39) (2023)	No	Unclear		Yes		Yes		Yes			No		Unclear	Unclear			NA		NA				NA
Kovacevich (40) (2023)	Yes	Yes		Yes		Yes		Yes			Yes		No	Yes			NA		NA				NA
Lyes (41) (2023)	No	Yes		Yes		Yes		Yes			Yes		Yes	Yes			NA		NA				NA
Lyons (42) (2022)	Yes	Yes		No		Yes		Yes			Yes		Yes	Yes			NA		NA				NA
Zarankin (38) (2024)	Yes	Yes		Yes		Yes		Yes			Yes		Yes	Yes			NA		NA				NA
JBI Critical Appraisal Checklist for Qualitative Research																							
Oblak (37) (2024)	Yes	Yes		Unclear		Unclear		Yes			Unclear		Yes	Yes			Yes			Yes			NA

RoB-2, Version 2 of the Cochrane Risk-of-Bias tool for randomized trials; ROBINS-E, Risk of Bias in Non-randomized Studies – of Exposures; ROBINS-I, the Risk-of-Bias in Non-Randomized Studies of Interventions; Q, question; JBI, Joanna Briggs Institute. * NA, JBI Critical Appraisal Checklist do not provide an overall appraisal score.

In the qualitative studies, four of the five case reports (80%) met most appraisal criteria, with consistent reporting of participant demographics, intervention details, outcomes, and follow-up (38, 40–42). In contrast, the fifth case report (20%) met substantially fewer JBI criteria, with multiple domains rated as no or unclear, particularly those related to participant history, clinical assessment, and outcome reporting (39). The mixed-methods qualitative study met most JBI criteria; however, several domains were rated as unclear, particularly those related to research positioning and analytic rigor (37). As JBI tools do not generate overall quality scores, qualitative study quality is reported descriptively rather than numerically. These appraisals reflect the quality of reporting rather than the strength of evidence for effect as case reports and case series describe selected individuals and cannot establish causal or efficacy relationships. The case reports were therefore interpreted as hypothesis-generating.

## 4 Discussion

This systematic review was conducted to examine and synthesize adult psilocybin MM or truffle microdosing, including reported health effects, perceived benefits, motivations or indications for use, dosing protocols and preparations, side effects and adverse events, differences by sex and age, while also exploring funding sources and competing interests. A total of 11 studies met inclusion/exclusion criteria and were included in this review.

Given the limited quantity of available evidence, the findings of this review should be interpreted cautiously. This synthesis characterizes what a small and methodologically heterogeneous body of literature reports; it does not establish whether microdosing psilocybin MM or truffles is effective or safe. The domain-specific findings discussed below should therefore be regarded as hypotheses requiring confirmation rather than as established effects.

### 4.1 Health effects

This narrative synthesis found that, across a small number of studies, some reported improvement from microdosing psilocybin MM or truffles in well-being, mental health, cognition, physical health, creativity, relationships, enhance senses, pain, curiosity, and substance dependence (37–41, 43, 44) among adults. These findings should be considered cautiously though, as the majority of the evidence had at least some concerns for risk of bias or was qualitative and used a variety of self-reported outcome measures and/or did not measure outcomes using valid and reliable instruments. Only one study analyzed findings based on sex, which did not find any significant difference in any outcomes (46), and none of the included studies investigated age differences.

This review’s findings align with existing research, which suggests that full-dose psilocybin combined with psychotherapy can improve mental health (3–7), certain domains of cognitive function (8, 9), social functioning (5, 8), creativity (8), and substance use (10). Our results are also consistent with one previous review on psychedelic microdosing for mental health (15). Conversely, these findings contrast with two other reviews on psychedelic microdosing (16, 17). These earlier reviews reported inconsistent findings (16) and decreased cognitive control (17) when examining various psychedelics combined and specifically found no benefit from microdosing psilocybin alone (17). A key difference is that previous systematic reviews investigating microdosing psychedelics often included any form of psilocybin, such as MM, truffles, and isolates. This approach is inconsistent with what is predominantly microdosed in community settings, which is mostly MM. Our current findings are important because they begin to establish evidence for the benefits of exclusively microdosing psilocybin MM and truffles, an area that has been lacking in the literature. To further strengthen this evidence, future RCTs should be conducted evaluating each type of psilocybin separately ensuring recruitment includes ethnically and racially diverse populations, various age groups, and a greater representation of women.

Of particular importance is the evidence from this review that microdosing psilocybin MM and/or truffles may be associated with improved mental health outcomes (37–39, 42–44) consistent with full-dose psilocybin findings (3–7) and one review microdosing combined psychedelic substances (15). However, the findings from this review were from studies that were mostly cross-sectional (43, 44) and qualitative (37–39, 42) designs, with the two clinical trials demonstrating no significant improvements (45, 46). It is important to highlight that the participants in the clinical trials were healthy adults and those with a history of mental health conditions and/or symptoms were excluded from the studies, whereas the participants in the cross-sectional and qualitative studies were microdosing MM or truffles for mental health conditions or symptoms. There are two potential explanations for these findings, either placebo and/or expectancy effects, which have been identified in the literature (4, 5, 7, 8, 10, 13–15), or a ceiling effect. Considering three studies included participants whose depression went into remission with depression scores reduced to zero and/or being able to stop antidepressant medications (38, 39, 42), it is likely that a ceiling effect may explain the null findings for the two trials (45, 46). It would be difficult to demonstrate statistically significant improvements in mental health outcomes if pretest scores in those outcomes were already normal to high. These findings are promising as a potential alternative treatment for mental health symptoms or conditions that could potentially be implemented in the community setting at lower costs than supervised full-dose psilocybin administration. However, future RCTs should be conducted to evaluate the effects of microdosing psilocybin MM with samples of participants who experience mental health symptoms using active placebos to ensure adequate blinding and minimize placebo and/or expectancy effects to confirm these findings.

This review provides limited evidence that microdosing psilocybin MM and truffles may enhance specific cognitive domains (36, 38, 43, 44). This aligns with previous research on full-dose psilocybin, which also found improvements in only certain cognitive functions (8, 9). Conversely, one systematic review on microdosing psychedelics reported decreased cognitive control (17), while another presented conflicting findings (16). Among the studies evaluating cognitive outcomes within this review, one yielded conflicting results (37) and two demonstrated no improvements (45, 46). The authors of the study with conflicting findings suspected participants were using larger doses than microdoses (37). Furthermore, one study with null findings noted that its unblinded microdosing group experienced a higher perceived intensity of acute effects compared to the control group (45). It is also important to note that three of these studies (two with null findings, one with conflicting) conducted post-testing assessments on the same day, within a few hours of administration (45, 46) or in real-time (37). This observation is consistent with prior psychedelic microdosing research that has reported impaired cognitive functioning as an adverse effect (16) and two systematic reviews on full-dose psilocybin that found acute interference related to the timing of product administration and post-assessments (8, 9). Collectively, these findings may suggest that microdosing psilocybin MM or truffles may only benefit certain cognitive domains, and/or post-test data collection could be influenced by acute effects. Both factors should be carefully considered in the methodological design of future intervention studies.

The three studies included in this review that did not find any health benefits from microdosing psilocybin MM and truffles were clinical trials testing the effects after a single dose (36), 2 doses over 1-week (45), and 5–7 doses over 3-weeks (46), with two studies (45, 46) testing impacts the same day as the dosing. On the contrary, four of the case studies that demonstrated health improvements reported participants chronically microdosed over 7-months (38), >1 year (41), 2-years (39), and 3-years (42), with only one case study including short-term microdosing, which was for three consecutive nights (40). Interestingly, the one case study with short-term microdosing improved an adult’s anosmia but mental health outcomes were not assessed in the study. This may suggest that to achieve desired health benefits from microdosing MM or truffles, especially mental health related outcomes, longer-term microdosing may be necessary, consistent with previous literature (13, 14).

Among the two studies that did not demonstrate health improvements (45, 46), one specifically examined psilocybin truffles for microdosing (46). Across all studies included in this review, only two exclusively used truffles (36, 46), and in one international observational study, only 6.6% of the sample reported using them (43). Furthermore, the suggested microdoses for psilocybin mushrooms and truffles differ, which may indicate distinct effects. The limited evidence evaluating psilocybin truffle microdosing currently prevents any definitive conclusions regarding its efficacy. Additionally, individual differences in adsorption rates of ingested mushrooms or truffles, especially when combined with food, supplements, vitamins, prescription drugs, and other substances may elicit a wide range of nervous system responses, and likely accounts for non-standardization of psilocybin amounts in a microdose. A personalized medicine approach along with nutrition and physical activity information collected for each participant before and after ingestion of their microdose MM samples would provide additional information as synergy and antagonism of natural products exist that can impact the circulating active dose of psilocybin (47). Moreover, research that combines findings from various microdosing substances hinders future progress in understanding their specific health effects (13, 15–17, 23). Therefore, it is crucial that all future research evaluating microdosing effects rigorously separates findings based on the psilocybin substance used; otherwise, advancements in this area will remain stagnant.

The generalizability of these findings is further limited by who was studied. Previous literature has emphasized the need for greater diversity of participants in psilocybin research, including sex, race, ethnicity, and age (3, 4, 6–10). Similarly, this review reflects the same gap demonstrating a pooled sample that was 70% male, and sociodemographic characteristics beyond sex and age were not largely reported across studies. Historically speaking females have been underrepresented in clinical trials and behavior-based research studies compared with males, hence the 30% female inclusion in these studies was not unexpected. According to a retrospective cohort review that evaluated female representation in non-prescription drug use clinical trials conducted in 2010-2019, only 35% of participants in trials targeting illicit drug use disorder were female (48). Females may have been excluded from participating in clinical and behavioral research due to policies and fear of hormonal fluctuations that may impact the data being collected. In addition to socioeconomic barriers that impede both males and females from participating in studies, as women tend to bear more of the caregiving responsibilities, they may also choose to forgo participation in studies that involve rigid schedules and multiple visits to clinical sites. Without sociodemographic information, it is not possible to determine whether findings generalize across populations, particularly given that cultural context, healthcare access, and attitudes toward psychedelics vary considerably across the countries represented in this review. For example, Latin American populations have distinct historical and cultural relationships with psilocybin that may shape how participants experience, interpret, and report microdosing effects (49). Also, access to conventional mental healthcare differs substantially across countries and may drive individuals toward microdosing as an alternative (15, 16). Legal status and social stigma surrounding psychedelic use vary widely across settings in ways that likely influence both who microdoses and how they describe the experiences (50). Future studies should prioritize demographic diversity, including sex, race, ethnicity, socioeconomic status, and geographic region, and should report these characteristics consistently to enable more generalizable and equitable conclusions from the microdosing literature.

### 4.2 Indications for use

Approximately 86% of microdoser motivations for using MM or truffles were related to non-medical diagnoses, including curiosity (43, 44), well-being (37, 43, 44), enhanced creativity (44), improved relationships (44), cognition (37, 43, 44), avoid bad habits (43, 44), and physical health (37, 43). The remaining 14% were microdosing MM or truffles for mental health (37, 38, 42–44), chronic pain (41), post-COVID anosmia (40), and Lyme disease symptoms (39). This may imply that overall, most individuals are microdosing for more holistic reasons. For this 14% of individuals microdosing to treat medical conditions, they may be choosing MM and truffles because they do not have access to adequate medical treatments or avoid or have been unsuccessfully treated with evidence-based treatments and are resorting to treatment options that have not yet been deemed efficacious or safe. Future studies should explore the healthcare access barriers driving medical microdosers toward MM and truffles and the rationale behind those microdosing for more holistic enhancement.

### 4.3 Dosing protocols and preparations

There was extreme heterogeneity across psilocybin MM and truffle preparations, microdosing, and scheduling within and across studies, preventing conclusions to be made regarding microdosing best practices. In addition, most studies did not justify or provide rationale for the preparation or dosing schedules, including the trials testing truffles (36, 46). Previous research has consistently indicated microdosing protocols have been extremely heterogeneous, lacking specific details, and/or not informed by the scientific literature (13, 15–17, 23), consistent with the findings of this review. Moreover, a precise dosage that reliably produces a sub-perceptual experience, which is fundamental to microdosing, remains undefined (23). To enhance the rigor of psilocybin microdosing research, it is essential to develop distinct dosing protocols. Specifically, determining separate dosages for psilocybin MM and truffles that elicit only sub-perceptual effects is crucial. Establishing these precise, separate protocols will facilitate more accurate comparisons across studies and enable robust meta-analyses in future systematic reviews.

### 4.4 Side effects and adverse events

A significant concern in the current literature is that approximately 64% of studies (36, 39, 40, 43, 45, 46) did not report any side effects or adverse effects experienced by participants, including those conducted as clinical trials (36, 45, 46). Of the remaining studies, only three reported both side effects and adverse events (37, 38, 41), highlighting the inconsistent and fragmented nature of safety reporting in this area. This omission is especially troubling given the lack of an established safety profile for microdosing psilocybin MM and truffles, and the consistent emphasis on explicitly reporting adverse effects in previous research (11, 13, 14, 16). Psilocybin is classified as a Schedule I substance by the U.S. Drug Enforcement Administration (51), “defined as drugs with no currently accepted medical use and a high potential for abuse,” and lacks U.S. Food and Drug Administration approval for use which further highlights the critical need for safety data. While the National Institutes of Health supports research into psilocybin for mental health and substance abuse, it does not currently back microdosing studies due to insufficient safety and preliminary efficacy data (52). Therefore, establishing the safety of psilocybin microdosing is a fundamental responsibility for all researchers in this area, and standardized reporting of adverse effects must become a mandatory practice.

It is important to note that the majority of reported side effects and adverse effects in the included studies were mild (41, 42, 44) consistent with previous literature (7, 10, 15, 16). However, one study (37) presented a notable exception, detailing intense anxiety symptoms, panic attacks, psychosis symptoms, suicidal ideation, and Crohn’s disease exacerbation among participants. Crucially, the authors of this study explicitly stated there was evidence these participants were not adhering to microdosing protocols (37). Consequently, these specific findings should be excluded from any future safety profiles to ensure an accurate representation of psilocybin microdosing risks.

### 4.5 Funding sources and potential conflicting interests

Four studies did not identify their funding sources and all four demonstrated improvements in outcomes (37–39, 41), and of the three studies that reported conflicts of interest, all three also demonstrated improvements (40, 41, 44). While these patterns alone are insufficient to draw conclusions about the influence of conflicts of interest on study outcomes, the lack of consistent funding disclosures across studies makes it impossible to properly evaluate this relationship. This is a significant concern in an emerging field where financial interests are growing rapidly and where the integrity of safety and efficacy reporting is critical. Across research fields, industry sponsorship has been consistently associated with outcomes favorable to the sponsor (53), and psychedelic research is not exempt from these concerns (54). Conflicts of interest in psychedelic research may manifest in multiple ways, including research topic selection biased toward commercially viable outcomes, selective screening of participants to increase the likelihood of positive results, and under-reporting of adverse events, with safety outcomes reporting in psychedelic research shown to be particularly vulnerable to stakeholder influence (54). Critically, the potential for conflicts of interest to contribute to publication bias and inaccurate adverse event reporting is of particular concern in this emerging field, where safety data remain limited and where positive findings may prematurely influence public perception and policy (11). Future research must enforce rigorous conflict of interest disclosure policies, consistent with standards applied across biomedical research, and funding agencies should support independent, government-funded studies to provide a counterbalance to commercially motivated research agendas.

### 4.6 Strengths

There are several strengths of this systematic review. This was the first review investigating the benefits of psilocybin MM or truffles exclusively, which are the most common forms of psilocybin microdosed in community settings. The sample of participants was large with participants living in several countries. We included articles published in English and Spanish which is important given that a meaningful portion of psilocybin microdosing is in Latin American countries (55), and excluding literature published in Spanish would have introduced language bias and narrowed the geographic representativeness of the review. However, these strengths concern the conduct of the review and should not be mistaken for strength of the underlying literature, which remains low.

### 4.7 Limitations

A meta-analysis was not conducted because of the substantial heterogeneity across study designs, dosing schedules, and reported outcomes (56, 57). In addition, the limited use of valid and reliable outcome measures further complicated both the narrative synthesis of study findings and the ability to draw any firm conclusions. The quantitative studies had concerns for bias and there were several qualitative studies included, limiting the strength of overall review findings. Five of the eleven included studies were single-patient case reports or small case series, which cannot establish efficacy and are prone to selective reporting. When these are set aside, the reported benefits rest almost entirely on uncontrolled cross-sectional and survey data, while the two placebo-controlled trials found no significant effect. Conclusions about benefit should therefore be regarded as preliminary and hypothesis-generating.

## 5 Conclusion

This systematic review is the first to exclusively synthesize evidence on the health effects of microdosing psilocybin MM and truffles among adults, representing an important contribution to a literature that has largely relied on anecdotal and non-scientific sources, particularly given the increasing accessibility and in-home use of psilocybin microdosing. The available evidence, however, is quantity, methodologically heterogeneous, and at high or unclear risk of bias. And is therefore insufficient to determine whether microdosing psilocybin MM or truffles is effective or safe. Although several studies reported improvement across multiple health domains, these findings derived predominantly from qualitative and cross-sectional designs with high or unclear risk of bias. Notably, placebo-controlled trials did not demonstrate significant improvements. A critical gap remains in the systematic reporting of adverse events, particularly given the chronic nature of community microdosing and the absence of an established long-term safety profile for psilocybin MM and truffles. To advance this field, future research should prioritize well-powered RCTs enrolling adults with clinically meaningful symptoms, standardized and substance-specific dosing protocols, mandatory adverse event reporting, and recruitment of demographically diverse samples.

## Data Availability

The original contributions presented in the study are included in the article/supplementary material. Further inquiries can be directed to the corresponding author/s.
